# The Role of Steroid Hormone Receptors in Urothelial Tumorigenesis

**DOI:** 10.3390/cancers12082155

**Published:** 2020-08-04

**Authors:** Hiroki Ide, Hiroshi Miyamoto

**Affiliations:** 1 Department of Urology, Keio University School of Medicine, Tokyo 160-8582, Japan; h-ide@fc4.so-net.ne.jp; 2 Department of Pathology & Laboratory Medicine, University of Rochester Medical Center, Rochester, NY 14642, USA; 3 Department of Urology, University of Rochester Medical Center, Rochester, NY 14642, USA; 4 James P. Wilmot Cancer Institute, University of Rochester Medical Center, Rochester, NY 14642, USA

**Keywords:** androgen receptor, estrogen receptor, glucocorticoid receptor, progesterone receptor, urothelial cancer, vitamin D receptor

## Abstract

Preclinical and/or clinical evidence has indicated a potential role of steroid hormone-mediated signaling pathways in the development of various neoplastic diseases, while precise mechanisms for the functions of specific receptors remain poorly understood. Specifically, in urothelial cancer where sex-related differences particularly in its incidence are noted, activation of sex hormone receptors, such as androgen receptor and estrogen receptor-β, has been associated with the induction of tumor development. More recently, glucocorticoid receptor has been implied to function as a suppressor of urothelial tumorigenesis. This article summarizes and discusses available data suggesting that steroid hormone receptors, including androgen receptor, estrogen receptor-α, estrogen receptor-β, glucocorticoid receptor, progesterone receptor and vitamin D receptor, as well as their related signals, contribute to modulating urothelial tumorigenesis.

## 1. Introduction

Urinary bladder cancer remains a commonly diagnosed malignant disease. The numbers of new bladder cancer cases and cancer deaths throughout the world have even risen from 429,800 and 165,100 estimated in 2012 [1] to 549,393 and 199,922 reported in 2018 [2], respectively. In addition to the bladder (and urethra), urothelial carcinoma occurs in the upper urinary tract (UUT) consisting of the renal calyces, renal pelvis and ureter. Although the incidence of UUT cancer is relatively low accounting for only 5–10% of urothelial carcinoma, approximately 60% of the cases (vs. 15–25% of bladder cancer) are invasive at the time of diagnosis [3]. Meanwhile, non-invasive urothelial carcinomas, particularly those in the bladder, are not usually lethal and can often be managed by conservative approaches including transurethral surgery. However, a considerable number of these patients still suffer from recurrent disease even following currently available intravesical pharmacotherapy. Thus, key molecules or signaling pathways responsible for the development of urothelial cancer need to be identified, which may subsequently provide targeted therapy options that more effectively prevent tumor recurrence.

Steroid hormones that usually function by binding to specific intracellular receptors are necessary for a variety of critical physiological processes. They have also been shown to contribute to the development of pathologic conditions, including neoplastic diseases. Specifically, emerging evidence suggests a vital role of the steroid hormone-mediated signaling pathway in both of two distinct events, urothelial carcinogenesis and cancer progression. In this article, we mainly review available preclinical and clinical data suggesting the involvement of the steroid hormone receptor superfamily, such as androgen receptor (AR), estrogen receptors (ERs), glucocorticoid receptor (GR), progesterone receptor (PR) and vitamin D receptor (VDR), in the pathogenesis of urothelial cancer. Moreover, we highlight several molecules whose expression and/or activity are not only modulated by steroid hormone receptor signals in urothelial cells but also directly involved in tumorigenesis.

## 2. AR

There have been constant sex-related differences, especially in the incidence of bladder cancer. Specifically, global cancer statistics data have shown a >3-fold higher risk of developing bladder cancer in men than in women [1,2]. Considering the male dominance, the involvement of AR signaling, as an intrinsic factor, in urothelial carcinogenesis has been explored, in addition to extrinsic risk factors such as cigarette smoke and industrial chemicals. Indeed, a retrospective study involving 1334 men with prostate cancer showed that the incidence of subsequent bladder cancer was significantly lower in those treated with androgen deprivation therapy (0/266, 0%), compared to those with radiotherapy (14/631, 2.2%) or prostatectomy (5/437, 1.1%) [4]. In addition, two other retrospective studies have assessed the impact of androgen deprivation therapy primarily for prostate cancer on the recurrence of non-muscle-invasive bladder cancer [5,6]. In men with a history of both prostate and bladder cancers, androgen deprivation therapy patients (*n* = 86) had a significantly lower risk of bladder cancer recurrence, compared with control patients without hormonal therapy (*n* = 76) (5-year recurrence-free survival: 76% vs. 40%, *p* < 0.001) [5]. In the androgen deprivation cohort for which tissue specimens were available (*n* = 72), AR expression in de novo bladder tumors, as an independent prognosticator, was further associated with successful prevention of tumor recurrence (hazard ratio (HR) = 0.27, *p* = 0.005) [7]. Similarly, men with androgen suppression therapy (i.e., androgen deprivation therapy for prostate cancer, 5α-reductase inhibitor treatment for benign prostatic hyperplasia) (*n* = 32) were found to have a significantly lower risk of bladder cancer recurrence, compared to those without androgen suppression therapy (*n* = 196) (multivariate analysis: HR = 0.36, *p* = 0.024) [6]. Thus, clinical evidence has indicated that androgen suppression prevents the development of bladder cancer.

AR expression has been assessed in surgical specimens, mostly using immunohistochemistry. Table 1 summarizes the findings of immunohistochemical studies, using both non-neoplastic and neoplastic tissues [8,9,10,11,12,13]. Some of these identified immunoreactivity for AR in non-neoplastic urothelium from 58–86% of cases and in bladder urothelial tumor from 42–53% of cases [8,9,11]. Similarly, AR signals were detected in 20% of UUT tumors versus 58% of corresponding normal-appearing urothelial tissues [13]. Thus, the expression of AR was significantly down-regulated in urothelial tumors, compared with non-neoplastic urothelial tissues. Indeed, in a recent study assessing the distribution of AR in the non-neoplastic lower urinary tract, AR was found to be ubiquitously expressed within the urothelium, with a marginal increase in the prostatic urethra [14]. However, two studies showed no AR expression in non-neoplastic urothelium, while AR was expressed in 51% [10] and 22% [12] of bladder tumors. Then, our meta-analysis of 5 immunohistochemical studies in bladder specimens demonstrated no significant difference in AR positivity between non-neoplastic and neoplastic tissues (*p* = 0.336) [15]. In addition, the expression of *AR* gene has been studied in non-muscle-invasive bladder cancer specimens, showing an association between higher mRNA levels and significantly lower risks of disease recurrence following transurethral surgery [16,17]. Of note, no studies have shown significant differences in the levels of AR mRNA/protein expression between urothelial tissues (i.e., benign urothelium, urothelial tumor) from male versus female patients, while the receptor activities that are closely associated with androgen levels have never been compared.

Alternations within the *AR* gene have also been documented in bladder cancer. The number of polyglutamine (CAG) repeats within exon 1 of the *AR* gene, which is usually associated inversely with its transcriptional activity, was found to be shorter in bladder tumors or patients with bladder cancer than in respective controls [18,19]. Shorter CAG repeats have also been associated with a significantly enhanced risk of bladder cancer (odds ratios (ORs) 2.09 in men and 4.94 in women) [18]. Meanwhile, western blotting in surgical specimens showed potential AR isoforms implying the presence of its splice variant(s) in bladder cancer [20]. In addition, analysis of molecular profiling data has suggested somatic mutations in the *AR* gene in a subset (e.g., 4–6.1%) of urothelial cancers [21].

Animal experiments have been employed to investigate the role of androgen-mediated AR signaling in urothelial tumorigenesis. In most of these studies, a bladder carcinogen *N*-butyl-*N*-4-hydroxybutyl nitrosamine (BBN), which reliably induces bladder tumors, especially in male rodents, has been used along with androgen/anti-androgen treatment and/or AR knockdown. An earlier study demonstrated that testosterone increased the incidence of bladder tumors in female rats treated with BBN, compared to those without testosterone (27.3% vs. 9.1%) [27]. A subsequent study showed that surgical or chemical castration or AR antagonist flutamide treatment reduced the occurrence of bladder cancer in BBN-treated male rats, while no combination effect was observed [28]. In addition, a 5α-reductase inhibitor finasteride did not have an impact on bladder cancer development, suggesting that the potency of testosterone vs. 5α-dihydrotestosterone (DHT) for promoting bladder tumorigenesis was similar. Early castration at 4 weeks of age has also been shown to prolong the survival of BBN-treated mice, compared with controls (315.8 days vs. 254.6 days, *p* < 0.05) [29]. In a study using AR knockout (ARKO) mice, BBN completely failed to induce bladder cancer by 40 weeks [30]. Interestingly, bladder cancer developed in 50% and 25% of castrated male mice and ARKO male mice supplemented with DHT [30], suggesting the involvement of AR pathway activated by non-androgens or low levels of androgens and non-AR pathways mediated by androgens, respectively, in urothelial carcinogenesis. More specifically, the incidence of bladder cancer was significantly lower in male mice lacking AR only in urothelial cells than in wild-type littermates [31], suggesting a critical role of urothelial AR in bladder tumorigenesis. More recently, a higher incidence of BBN-induced bladder tumors was found in transgenic male (67% vs. 23%) and female (39% vs. 0%) mice where AR was conditionally expressed in the bladder, compared with respective control littermates [32].

An *in vitro* transformation system using non-neoplastic SVHUC urothelial cells with carcinogen challenge has also been applied as a model of cancer initiation. When compared between SVHUC and SVHUC-AR expressing full-length human AR upon exposure to a chemical carcinogen 3-methylcholanthrene (MCA), overexpression of AR was found to accelerate the neoplastic transformation of urothelial cells [33,34,35]. Similarly, androgen treatment resulted in the induction of the neoplastic formation of MCA-SVHUC-AR cells [33]. Correspondingly, three anti-androgens clinically used for the treatment of prostate cancer, including bicalutamide, hydroxyflutamide and enzalutamide, showed similar inhibitory effects on the neoplastic transformation of MCA-SVHUC-AR cells [34].

The current preclinical findings thus indicate that AR activation is associated with promotion of urothelial tumorigenesis, which is also supported by retrospective studies described above [4,5,6,7] suggesting the inhibitory effect of androgen deprivation therapy on the development and recurrence of bladder tumor. Indeed, early phase clinical trials have been conducted to assess the efficacy of AR antagonists in, for instance, the prevention of bladder tumor recurrence after transurethral surgery.

## 3. ERs

There are two distinct forms of the ER, ERα and ERβ, which are encoded by *ESR1* and *ESR2*, respectively. The transcriptional activity of ERα and ERβ can be differentially induced by certain ligands in a cell-type- or promoter-specific manner. They also exhibit distinct tissue/cell-type-specific expression patterns. An immunohistochemical study in human bladder tumors indeed showed that the rates of ERα and ERβ positivity were approximately 1% and 63%, respectively [36].

The expression of ERα and ERβ has been immunohistochemically compared in bladder cancer specimens versus corresponding benign tissues (Table 1). The rates of ERα positivity were shown to be significantly higher in non-neoplastic urothelium than in tumor [11], while other studies showed no significant differences in ERα expression between non-neoplastic and neoplastic tissues [12,22]. Our recent meta-analysis of immunohistochemical studies in bladder specimens also revealed a significant difference in ERα expression between non-tumor and tumor (*p* < 0.001) [15]. Similarly, several studies stained for ERβ in bladder cancer samples showed significantly higher positivity in non-tumors than in tumors [11,23]. However, our meta-analysis failed to show significant down-regulation of ERβ expression in bladder tumors (*p* = 0.674) [15]. In the nephronureterectomy specimens with UUT urothelial carcinoma, ERα/ERβ expression was significantly down-regulated in non-neoplastic urothelial tissues, compared with carcinoma tissues [13]. By contrast, quantitative PCR data showed considerable increases in ERα expression, but not ERβ expression, in bladder tumors, compared with non-neoplastic urothelial cells [37]. Similar to AR expression, no significant differences in ERα or ERβ mRNA/protein expression between male and female urothelial tumors have been reported, while no studies have compared the actual receptor activities. Recently, epigenetic alternations, including *ESR* methylation, have been investigated in bladder cancer, because methylation seems to be an early event in the development of solid tumors [38]. In a study showing the methylation status of 21 genes in bladder specimens, 44% of tumors vs. 20% of non-tumors were found to have *ESR* methylation (*p* = 0.622) [39].

Studies using preclinical models for urothelial cancer have been performed to determine the role of estrogen-mediated ER signaling in bladder carcinogenesis. Two early studies in female rodents showed that bilateral ovariectomy (30%) increased the incidence of BBN-mediated bladder tumors, compared with controls (18%) [27], while 17β-estradiol (E2) treatment resulted in the regression of transplanted bladder tumors [40], suggesting the preventive effects of estrogens on urothelial cancer outgrowth. This was further supported by the findings in the former study [27] demonstrating that bladder cancer incidence was significantly lower in male rats treated with a synthetic estrogen diethylstilbestrol (also inducing chemical castration in males) (7%) than in those undergoing surgical castration only (50%). By contrast, female mice prenatally exposed to arsenic, which was known to be a strong bladder carcinogen and shown to induce the expression of ERα as well as other estrogen-regulated molecules, followed by postnatal diethylstilbestrol treatment (48%) significantly more often developed malignant urogenital tumors, compared to those with arsenic exposure alone (9%), suggesting the oncogenic role of estrogen/ERα in urogenital carcinogenesis [41]. Using mouse gene knockout models treated with BBN, bladder cancer was shown to be induced significantly more or less often in ERα knockout females (81%) [vs. wild-type female littermates (46%)] [42] or in ERβ knockout females (23%) [vs. wild-type female littermates (75%)] [43] respectively. Thus, ERα and ERβ appear to show inhibitory and stimulatory effects, respectively, on urothelial tumorigenesis. Another study showed a significantly lower incidence of bladder cancer induced by BBN in female mice concurrently (14%; all non-muscle-invasive) or concurrently + subsequently (10%; 7% non-muscle-invasive and 3% muscle-invasive) treated with an anti-estrogen tamoxifen than in controls (76%; 55% non-muscle-invasive and 21% muscle-invasive) [44]. Interestingly, ERα was immunohistologically detected in bladder tissues from none of the control mice without BBN exposure but 74% of BBN-treated mice, indicating a possible role of ERα in inducing urothelial carcinogenesis, while ERβ was expressed in all these animals with or without BBN [44].

## 4. GR

GR, as two major alternative isoforms, GRα and GRβ, is expressed in virtually every cell in the human body. GRα, as the classic GR, mediates the actions of glucocorticoids, leading to the regulation of the glucocorticoid response element-mediated transcription of genes (i.e., transactivation) as well as the activity of other transcription factors, such as AP-1 and NF-κB, on the target genes (i.e., transrepression) [45]. By contrast, deletion of the unique C-terminal amino acids in GRβ prevents it from binding glucocorticoids or activating glucocorticoid-responsive promoters [46]. Although GRβ was described as a dominant-negative inhibitor of GRα [47], its function has not been well understood.

Our immunohistochemical staining in bladder [24] and UUT [13] samples detected the expression of GR in non-neoplastic urothelial tissues in most of the cases, which was significantly down-regulated in urothelial neoplasms (Table 1). In addition, loss of strong GR expression in non-muscle invasive bladder tumors, as an independent predictor (HR = 2.252; *p* = 0.034), was associated with a significantly higher risk of disease recurrence [24]. These immunohistochemical data in surgical specimens suggest that GR signals contribute to preventing the development of urothelial cancer.

Glucocorticoids are a class of medications prescribed for the treatment of various inflammatory and autoimmune disorders, but certain drugs, such as dexamethasone and prednisone, have also been given, as cytotoxic agents, to patients with, for instance, hematological malignancy or castration-resistant prostate cancer [48]. Meanwhile, it has been documented that prolonged systemic use of glucocorticoids use was at an increased risk of developing bladder cancer [49], presumably due to associated immune suppression. Our recent studies using the *in vitro* transformation system described above demonstrated that GR knockdown in SVHUC cells resulted in the significant prevention of MCA-induced neoplastic transformation of urothelial cells [50]. More interestingly, of a total of 11 glucocorticoids screened including dexamethasone, only prednisone significantly inhibited the neoplastic transformation of urothelial cells. Moreover, the preventive effects of prednisone on the neoplastic transformation of GR-positive control SVHUC cells were considerably diminished by a GR antagonist RU486, while prednisone failed to significantly affect the neoplastic transformation of GR knockdown cells. Correspondingly, in a BBN mouse model, prednisone (50%) prevented the development of bladder cancer at 18 weeks of age, compared with mock (100%; *p* = 0.021) or dexamethasone (87.5%) treatment. Additional experiments in SVHUC cells revealed that dexamethasone could induce both transactivation and transrepression of GR, while prednisone preferentially induced GR transrepression. These findings suggest that glucocorticoid-mediated GR signals prevent urothelial tumorigenesis primarily via transrepression.

Compound A [CpdA; 2-(4-acetoxyphenyl)-2-chloro-*N*-methyl-ethylammonium chloride] is a unique chemical substance which has been known to function as an AR antagonist as well as a GR ligand [51]. We have additionally demonstrated that CpdA induces only GR transrepression in SVHUC cells [52] and bladder cancer lines [53]. In SVHUC-AR cells expressing both GR and AR upon carcinogen challenge, CpdA inhibited their neoplastic transformation and its effect was stronger than prednisone or hydroxyflutamide [52]. CpdA and prednisone showed similar inhibitory effects on the neoplastic transformation of AR-negative MCA-SVHUC cells, which was antagonized by RU486, but no significant inhibition in AR-negative/GR-negative cells. The preventive effects of CpdA on bladder tumorigenesis were further confirmed in male mice exposed to BBN along with mock (100%), CpdA (25%; *p* = 0.002 vs. mock), prednisone (50%) or flutamide (50%) treatment [52].

## 5. PR

Progesterone is the major progestogen which plays a critical role in the menstrual cycle and pregnancy and is also used for pharmacological female contraception and postmenopausal hormone therapy. In the UPII-SV40T transgenic model where bladder cancer was spontaneously developed, tumor size was significantly smaller in multiparous female mice than in nulliparous females (*p* < 0.001), implying that progesterone exhibited a protective effect on urothelial cancer outgrowth [54]. Similarly, in a case-control study involving 779 Egyptian women, oral contraceptive use (adjusted OR = 0.44; *p* < 0.0001), multiple (>7) pregnancies (adjusted OR = 0.66; *p* = 0.08) and multiple (>6) deliveries (unadjusted OR = 0.7; *p* = 0.07) were associated with lower risks of bladder cancer [55].

Immunohistochemical studies showed the rates of PR positivity were <5% and 13–16% in bladder and UUT specimens, and their differences between non-neoplastic urothelium and urothelial tumor were not statistically significant [12,13,22] (Table 1). However, no functional studies of PR signals in urothelial cancer have been reported, except for recent analysis of microarray datasets showing that *PR* gene was differentially expressed in normal urothelial tissues versus urothelial carcinomas [56].

## 6. VDR

Vitamin D is a group of fat-soluble secosteroids. As shown in a meta-analysis (pooled relative risk = 0.75, *p* < 0.001) [57], low serum levels of 25-hydroxyvitamin D have been implicated in a higher risk of bladder cancer. A case-control study involving 130 bladder cancer patients versus 346 normal individuals also showed significant differences in the genotype (OR for “FF” = 2.042) or allelic frequency (OR for “F” = 1.489) of VDR (Fok-l) polymorphism [58], suggesting the association between reduced VDR activity and elevated bladder cancer risk. Furthermore, using rats treated with a carcinogen N-methylnitrosourea, intravesical administration of vitamin D was found to reduce the incidence of bladder cancer (55%), compared with mock-treated rats (66%). Notably, the rate of invasive tumors in those treated with vitamin D (20%) was significantly lower than that with mock treatment (50%) [59]. These findings suggest that VDR functions as a tumor suppressor and its activation prevents urothelial tumorigenesis as well as tumor progression.

Immunohistochemical studies in surgical specimens have determined the status of VDR expression in non-neoplastic and neoplastic urothelial tissues (Table 1). The one revealed significant up-regulation of VDR expression in bladder tumors [25], whereas the other showed no significant difference in VDR positivity [59]. Additionally, in the latter study [26], the expression of CYP27B1, which could contribute to producing a biologically active form of vitamin D, was shown to be significantly (*p* = 0.03) down-regulated in tumors, compared with normal urothelial cells.

## 7. Molecules Modulated by Steroid Hormone Receptor Signaling in Urothelial Cells

**Figure 1:**
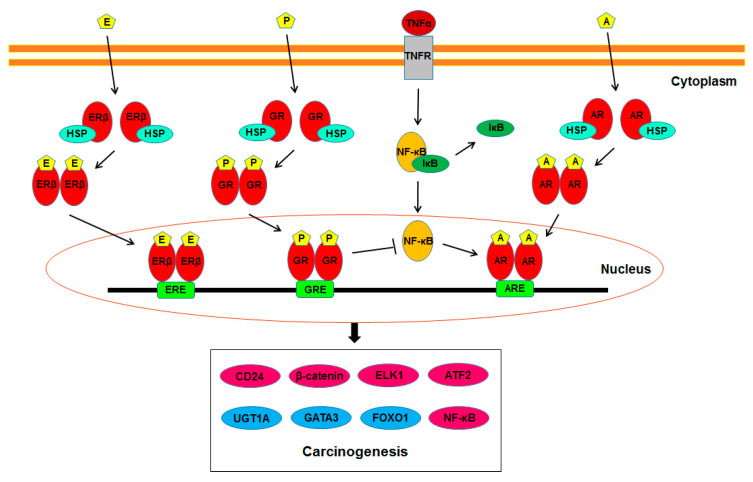
AR/ERβ/GR signaling in urothelial carcinogenesis. A, androgen; AR, androgen receptor; ARE, androgen response element; E, estrogen; ER, estrogen receptor; ERE, estrogen response element; GR, glucocorticoid receptor; GRE, glucocorticoid response element; HSP, heat shock protein; P, prednisone; TNF, tumor necrosis factor; TNFR, tumor necrosis factor receptor.

As described above, increasing evidence suggests the involvement of steroid hormone receptor-mediated signals in the development of urothelial cancer. Further studies have identified their potential downstream targets that may play a vital role in modulating urothelial tumorigenesis. Figure 1 summarizes such molecules directly or indirectly regulated by AR, ERβ, and/or GR signals. The following are key molecules whose expression and/or activity have been shown to be modulated via the androgen-AR/estrogen-ERβ/glucocorticoid-GR signaling pathways in non-neoplastic urothelial (or bladder cancer) cells.

### 7.1. UDP-Glucuronosyltransferases (UGTs)

UGTs are a family of drug metabolism enzymes responsible for catalyzing the glucuronidation of carcinogenic compounds. Of the family members, several UGT1A subtypes are known to play a critical role in detoxifying bladder carcinogens, such as aromatic amines and those derived from cigarette smoke. Correspondingly to a potential difference in the susceptibility to bladder carcinogens between men and women, the expression levels of mouse *Ugt1a* subtypes were shown to be considerably higher in the bladders from females than in those from males [60]. In immunohistochemical studies in bladder [61] or UUT [62] specimens, UGT1A expression was down-regulated in tumors, compared with non-neoplastic urothelial tissues, as well as in high-grade and/or muscle-invasive tumors, compared with low-grade and/or non-muscle-invasive tumors, and its positivity was associated with better patient outcomes.

In the normal urothelial cell line SVHUC, overexpressing of human wild-type AR resulted in the considerable down-regulation of UGT1A expression [60]. In SVHUC-AR cells, DHT considerably reduced the expression levels of *UGT1A* subtypes as well as UGT1A protein [60]. Moreover, bilateral orchiectomy in male mice up-regulated the expression of some *Ugt1a* subtypes in their bladders, which was restored by DHT supplement [60]. The levels of some *Ugt1a* subtypes were also significantly higher in the bladders from ARKO male mice than in those from wild-type littermates, while DHT supplement in these ARKO mice did not reduce their expression [60]. Similarly, E2 treatment induced UGT1A expression at both mRNA and protein levels in SVHUC cells endogenously expressing ERβ, while bilateral ovariectomy in female mice reduced *Ugt1a* expression in their bladders [61]. In addition, castration in male mice was found to reduce bladder susceptibility to a carcinogen 4-aminobiphenyl via modulating the activity of UGT1A in the liver [63]. Meanwhile, in the in vitro transformation system with MCA exposure, *UGT1A* expression was significantly down-regulated and up-regulated by GR knockdown [48] and hydroxyflutamide/prednisone/CpdA treatment [52], respectively. Our immunohistochemical study in bladder tumors showed that the expression of UGT1A was positively and negatively correlated with those of ERα and ERβ, respectively [61]. Thus, activation of AR and ERβ signals appears to be associated with the suppression and induction, respectively, of UGT1A expression in urothelial cells.

### 7.2. GATA3

GATA3 belongs to the GATA family of zinc-finger transcription factors and is known to involve the morphogenesis of some organs, such as the mammary gland and urogenital system. Indeed, in diagnostic surgical pathology, GATA3 immunohistochemistry has been widely used as a marker of urothelial differentiation [64]. In SVHUC cells subsequently exposed to MCA, GATA3 knockdown resulted in the promotion of neoplastic transformation, along with down-regulation of the expression of tumor suppressor genes (e.g., *p53*, *p21*, *PTEN*) and up-regulation of that of oncogenes (e.g., *c-myc*, *cyclins*, *FGFR3*) [33], suggesting its function as a suppressor of urothelial tumor. Immunohistochemical studies in bladder [65] or UUT [62] specimens further showed significant down-regulation of UGT1A expression in tumors, compared with non-neoplastic urothelial tissues.

In SVHUC-AR cells, androgens reduced GATA3 expression, which was blocked by hydroxyflutamide or bicalutamide [33]. By contrast, E2 treatment in ERα-negative/ERβ-positive SVHUC cells induced GATA3 expression, which was blocked by tamoxifen [33]. Additionally, in mouse bladders, orchiectomy in males and ovariectomy in females resulted in an increase and a decrease, respectively, in the expression of GATA3 [33]. Our immunohistochemistry data in bladder specimens further showed correlations between GATA3 expression versus AR overexpression, ERα overexpression or loss of ERβ expression [65], while, in UUT tumors, there were positive correlations of GATA3 with AR or ERβ but not ERα [66]. Thus, androgens and estrogens appear to reduce and induce GATA3 expression via the AR and ERβ (or ERα) pathways, respectively, in urothelial cells.

### 7.3. FOXO1

FOXO1 is a member of the forkhead transcription factor family, which has been demonstrated to modulate cellular functions such as cell cycle progression and apoptosis. FOXO1 can be inactivated by its phosphorylation through several protein kinases, including PI3K/Akt, resulting in the inhibition of cell growth. As suggested in several other types of malignancies, we recently demonstrated that FOXO1 knockdown or inhibitor treatment prevented the MCA-mediated neoplastic transformation of SVHUC cells [67], indicating its function as a suppressor for urothelial cancer. In addition, immunohistochemistry in bladder specimens showed significant down-regulation of FOXO1, as well as significant up-regulation of a phosphorylated inactive form (p-FOXO1), in urothelial tumors, compared with non-neoplastic urothelial tissues [67]. In UUT specimens, the levels of p-FOXO1 expression in tumors were also found to be significantly elevated, compared with corresponding benign tissues [68].

AR overexpression in SVHUC cells or DHT treatment in SVHUC-AR cells resulted in the reduction of the expression or transcriptional activity of FOXO1, as well as the induction of p-FOXO1 expression [67]. Similarly, ERβ knockdown induced the levels of FOXO1 mRNA/protein expression or transcription and reduced those of p-FOXO1 expression in SVHUC cells, while E2 treatment showed opposite effects [67]. Chromatin immunoprecipitation in bladder cancer cells further revealed that ERβ could bind to the FOXO1 promoter [67]. Immunohistochemistry showed significant correlations between p-FOXO1 expression and AR positivity in bladder tumors or ERβ positivity in UUT tumors, as well as between FOXO1 expression and ERα positivity or ERβ negativity [67,68]. These observations suggest that activation of AR or ERβ is associated with inactivation of FOXO1 signals in urothelial cells.

### 7.4. CD24

CD24 is a sialoglycoprotein and is thought to generally function as a cell adhesion molecule. It has also been described as a marker for bladder cancer stem cells and its expression levels were significantly higher in bladder tumors than in adjacent non-cancer tissues [69]. An association between elevated CD24 expression in non-muscle-invasive tumors and a significantly higher risk of disease recurrence has been reported [70]. In addition, the incidence of bladder cancer induced by BBN was significantly lower in CD24-deficient male mice (29%) than in wild-type controls (45%) at 16 weeks [71]. Elevated CD24 expression was also observed in bladder tumors from BBN-treated wild-type male and female mice, compared with normal urothelial tissues. Thus, CD24 appears to act as a driver of urothelial tumorigenesis.

The significant impact of CD24 knockdown in male mice on the rate of BBN-induced bladder cancer development was not seen in female mice (wild-type: 33% vs. CD24-deficient: 24% at 16 weeks) [71], implying the involvement of AR signaling in CD24-mediated urothelial tumorigenesis. In the same study, striking increases in CD24 expression by androgen treatment were observed in AR-positive bladder cancer lines [71], although no such changes in non-neoplastic urothelial cells have been demonstrated. Furthermore, binding of AR to the CD24 promoter at an AR-responsive element in bladder cancer cells was suggested [72].

### 7.5. β-Catenin

β-Catenin is a multifunctional protein and represents a key component of the canonical Wnt signaling pathway initially identified for its role in tumorigenesis. Downstream effectors of Wnt/β-catenin signaling include oncogenes, such as *c-myc*. Indeed, genetic alterations, aberrant expression, and/or activation of β-catenin as well as Myc in bladder cancer have been documented [73].

Using an inducible transgenic mouse model where β-catenin can be constitutively activated, castration in males was shown to reduce not only the incidence of bladder cancer but also AR expression in tumors [74], suggesting that androgen-mediated AR signals could enhance β-catenin-induced bladder tumorigenesis. In AR-positive bladder cancer cells, we demonstrated that androgens induced the nuclear expression of β-catenin and its interaction with AR, along with c-myc expression, and thereby activated the Wnt/β-catenin signaling pathway [75].

### 7.6. ELK1

ELK1 is an ETS family member and is known to activate, as a transcription factor, downstream targets, including a proto-oncogene *c-fos*. We demonstrated, using SVHUC cells with carcinogen challenge, that knockdown of ELK1 or treatment with a selective α_1_-blocker silodosin, which could inactivate ELK1, prevented the MCA-induced neoplastic formation of SVHUC-AR cells [35], indicating the oncogenic role of ELK1 in urothelial cancer. Our immunohistochemical studies in bladder [76] and UUT [77] specimens showed significant up-regulation of the expression of ELK1 and/or its activated form phospho-ELK1 in tumors, compared with non-neoplastic urothelial tissues. In addition, phospho-ELK1 positivity in non-muscle-invasive bladder tumors was associated with a significantly higher risk of disease recurrence [76].

AR overexpression in SVHUC cells or DHT treatment in SVHUC-AR cells resulted in the induction of ELK1 expression [35]. Interestingly, ELK1 inactivation via its knockdown or silodosin treatment failed to significantly affect the neoplastic transformation of urothelial cells lacking a functional AR [35]. Moreover, in bladder cancer lines, DHT and silodosin did not significantly induce and inhibit the proliferation of ELK1 knockdown cells and that of AR-negative cells or AR-positive cells cultured in an androgen-depleted condition, respectively [76,78]. In bladder tumor samples, the expression of AR and ELK1 or phospho-ELK1 was significantly correlated [76]. These findings suggest not only that the activities of AR and ELK1 are correlated but also that ELK1 requires an activated AR for functioning as an oncogenic molecule in urothelial cells.

### 7.7. ATF2

ATF2 is a member of the leucine zipper family of transcription factors and forms a homodimer or heterodimer with other family members, such as c-jun and c-fos. The activity of ATF2 is normally regulated via its phosphorylation through ERK/MAPK signals. Similar to the findings in ELK1, we demonstrated prevention of the neoplastic transformation of MCA-SVHUC-AR cells by ATF2 knockdown [79]. Immunohistochemistry in transurethral resection specimens also showed significant elevation of the expression of ATF2, phospho-ATF2 and phospho-ERK in bladder tumors, compared with non-neoplastic urothelial tissues [79].

The expression levels of ATF2 and phospho-ATF2 were considerably higher in SVHUC-AR than in AR-negative SVHUC [79]. In AR-positive bladder cancer cells, DHT induced the expression of phospho-ATF2 and phospho-ERK as well as nuclear translocation and transcriptional activity of ATF2. In bladder tumor specimens, significant correlations between immunoreactivities to AR versus ATF2 or phospho-ATF2 were observed.

### 7.8. NF-κB

NF-κB is a homo- or hetero-dimeric transcription factor complex and represents a key regulator of genes that control carcinogenesis (and tumor progression). Of the members that constitute the complex, RELA/p65, via its phosphorylation (and acetylation), plays a crucial role in post-translational modifications required for NF-κB activation. We recently showed that the expression of p65 and phospho-p65 was significantly elevated in bladder tumors, compared with corresponding benign urothelial tissues, and that the activity of NF-κB modulated by its activator or inhibitor was associated with urothelial tumorigenesis, using carcinogen-induced models (e.g., MCA in SVHUC cells, BBN in mice) [80].

Functional interplay between AR and NF-κB signals has been documented in, for instance, prostate cancer cells [81,82]. We additionally demonstrated that a pharmacologic activator/inhibitor of NF-κB induced/reduced, respectively, the expression and transcriptional activity of AR in non-neoplastic urothelial cells [80]. Similarly, in bladder cancer cells, DHT enhanced NF-κB transactivation, which was blocked by hydroxyflutamide [80]. Thus, AR and NF-κB signals are likely modulated by each other in urothelial cells. More interestingly, NF-κB activator/inhibitor failed to significantly affect the neoplastic transformation of urothelial cells lacking a functional AR [80], indicating that the presence of activated AR is necessary for modulating urothelial tumorigenesis via NF-κB. In bladder tumors, immunoreactivity to NF-κB versus AR was significantly correlated [80].

Besides possible direct interactions between AR and GR signals indicated in prostate cancer [83], NF-κB is known to inhibit GR activity via down-regulating the expression of target genes that are normally up-regulated by NF-κB [45]. Suppression of NF-κB transactivation thus represents an indirect mechanism of glucocorticoid action referred to as GR transrepression. As described above, GR transrepression induced by prednisone [50] and CpdA [52] is associated with the prevention of urothelial tumorigenesis.

## 8. Conclusions

Emerging evidence suggests that steroid hormone receptor-mediated signals play a critical role in urothelial tumorigenesis. Specifically, AR/ERβ and ERα/GR/PR/VDR may promote and prevent, respectively, the development of urothelial tumor, although conflicting findings exist. Several molecules have also been suggested to function as effectors for steroid hormone receptors in urothelial cells. In addition, although the functional interplay between steroid hormone receptor signals in urothelial cells has not been confirmed, some of the molecules, such as NF-κB, have been found to link multiple receptors. Further investigation of how steroid hormone receptor signals modulate urothelial tumorigenesis is required, which may subsequently help develop better strategies for the prevention of recurrent urothelial tumors or tumor development in otherwise high-risk populations.

## Figures and Tables

**Table 1:** Immunohistochemical studies for the expression of steroid hormone receptors in non-neoplastic urothelium versus urothelial carcinoma specimens.

Author, Year [Reference]	Receptor	Tumor Site	Positive/Total Cases		
			Non-Tumor	Tumor	*p* Value
Boorjian, 2004 [8]	AR	Bladder	32/37 (86%)	26/49 (53%)	0.001 *
Kauffman, 2011 [9]	AR	Bladder	50/59 (84%)	30/59 (51%)	<0.001
Tuygun, 2011 [10]	AR	Bladder	0/58 (0%) (Male)	71/139 (51%)	<0.001 *
Miyamoto, 2012 [11]	AR	Bladder	113/141 (80%)	79/188 (42%)	<0.001
Mashhadi, 2014 [12]	AR	Bladder	0/132 (0%)	26/120 (22%)	<0.001
Kashiwagi, 2015 [13]	AR	UUT	46/80 (58%)	20/99 (20%)	<0.001
Miyamoto, 2012 [11]	ERα	Bladder	70/141 (50%)	51/188 (27%)	<0.001
Mashhadi, 2014 [12]	ERα	Bladder	2/132 (2%)	3/120 (3%)	0.67
Kashiwagi, 2015 [13]	ERα	UUT	32/80 (40%)	18/99 (18%)	0.001
Imai, 2019 [22]	ERα	Bladder	33/92 (36%)	48/125 (38%)	0.777 *
Kontos, 2010 [23]	ERβ	Bladder	27/29 (93%)	84/111 (76%)	0.041 *
Miyamoto, 2012 [11]	ERβ	Bladder	125/141 (89%)	93/188 (49%)	<0.001
Kashiwagi, 2015 [13]	ERβ	UUT	68/80 (85%)	62/99 (63%)	0.001
Ishiguro,2014 [24]	GR	Bladder	90/94 (96%)	129/149 (87%)	0.026
Kashiwagi, 2015 [13]	GR	UUT	67/80 (84%)	62/99 (63%)	0.001
Mashhadi, 2014 [12]	PR	Bladder	3/132 (2%)	5/120 (4%)	0.48
Kashiwagi, 2015 [13]	PR	UUT	10/80 (13%)	16/99 (16%)	0.487
Imai, 2019 [22]	PR	Bladder	1/92 (1%)	4/125 (3%)	0.398 *
Sahin, 2005 [25]	VDR	Bladder	70/105 (67%)	90/105 (86%)	0.02
Jóźwicki, 2015 [26]	VDR	Bladder	12/12 (100%)	62/71 (87%)	0.345 *

AR: androgen receptor; ER: estrogen receptor; GR: glucocorticoid receptor; PR: progesterone receptor; VDR: vitamin D receptor; UUT: upper urinary tract. * We calculated the two-tailed *p* values using Fisher’s exact test.
